# Functional redundancy of transcription factors SlNOR and SlNOR-like1 is required for pollen development in tomato

**DOI:** 10.1093/hr/uhaf003

**Published:** 2025-01-06

**Authors:** Hong-Li Li, Lan-Ting Xiang, Xiao-Dan Zhao, Ben-Zhong Zhu, Hong-Liang Zhu, Gui-Qin Qu, Yun-Bo Luo, Ying Gao, Cai-Zhong Jiang, Da-Qi Fu

**Affiliations:** Laboratory of Fruit Biology, College of Food Science & Nutritional Engineering, China Agricultural University, No.17, Qinghua East Road, Haidian,Beijing, 100083, China; Laboratory of Fruit Biology, College of Food Science & Nutritional Engineering, China Agricultural University, No.17, Qinghua East Road, Haidian,Beijing, 100083, China; School of Food and Health, Beijing Technology and Business University, No.11, Fucheng Road, Haidian, Beijing, 100048, China; Laboratory of Fruit Biology, College of Food Science & Nutritional Engineering, China Agricultural University, No.17, Qinghua East Road, Haidian,Beijing, 100083, China; Laboratory of Fruit Biology, College of Food Science & Nutritional Engineering, China Agricultural University, No.17, Qinghua East Road, Haidian,Beijing, 100083, China; Laboratory of Fruit Biology, College of Food Science & Nutritional Engineering, China Agricultural University, No.17, Qinghua East Road, Haidian,Beijing, 100083, China; Laboratory of Fruit Biology, College of Food Science & Nutritional Engineering, China Agricultural University, No.17, Qinghua East Road, Haidian,Beijing, 100083, China; Key Laboratory of Plant Hormones and Development Regulation of Chongqing, School of Life Sciences, Chongqing University, No.174, Zheng Street, Shapingba, Chongqing, 400044, China; Department of Plant Sciences, University of California, One Shields Avenue, Davis, 95616, CA; Crops Pathology and Genetics Research Unit, United States Department of Agriculture, Agricultural Research Service, One Shields Avenue, Davis, 95616, CA; Laboratory of Fruit Biology, College of Food Science & Nutritional Engineering, China Agricultural University, No.17, Qinghua East Road, Haidian,Beijing, 100083, China

## Abstract

In tomato, SlNOR and SlNOR-like1*,* members of the NAC family of transcription factors (TFs), are known to play critical roles in regulating fruit ripening and are highly expressed in floral organs. However, their role in flower development remains unclear. In this study, we generated and functionally characterized a double knockout mutant, *nor/nor-like1*. Our findings reveal that the pollen abortion of the *nor/nor-like1* impedes ovarian enlargement, resulting in fruit formation failure. Histological analyses demonstrate that the pollen wall collapse occurs during the mature pollen stage and leads to the abnormal pollen wall component deposition at the microspore stage, resulting in the male sterility in the double knockout mutant lines. Kyoto Encyclopedia of Genes and Genomes enrichment pathway analyses further suggest that the loss of SlNOR and SlNOR-like1 function affects several metabolic pathways related to pollen development, including ‘ABC transporters’, ‘lipid metabolism’, ‘phenylpropanoid biosynthesis’, ‘hormone signal transduction’, ‘starch and sucrose metabolism’, and ‘cutin, suberine, and wax biosynthesis’. Furthermore, our results demonstrate that SlNOR and SlNOR-like1 could directly bind to the promoters of key genes associated with pollen wall formation and activate their expression, including ATP-binding cassette transporters of the G family (*SlABCG8/9/23*), ECERIFERUM (*SlCER1*), and glycine-rich protein (*SlGRP92*). These findings suggest that SlNOR and SlNOR-like1 may play a redundant role in the biosynthesis and transport of sporopollenin precursors, cuticular wax biosynthesis, and exine formation. In summary, our study highlights a previously uncharacterized role of *SlNOR* and *SlNOR-like1* in tomato pollen wall formation and male fertility.

## Introduction

Successful reproduction of flowering plants relies on the development and maturity of pollen. Mature pollen grains are released from the anther and dispersed by environmental agents such as wind and insects, eventually reaching the stigma of the pistil where they germinate and form pollen tubes that grow toward the ovule [1, 2]. Female gametophyte secretes a range of attractants to entice the pollen tube for fertilization while pollen tubes continuously alter their growth direction in response to this signal [3, 4]. Fertilization occurs when sperm cells from the pollen fertilize the egg cells in the ovule, leading to the formation of a plant embryo. In the post-fertilization process, the embryo develops into a seed while the ovary develops into a fruit. Failure to complete this process can result in male infertility, which is commonly observed in nature. In agricultural breeding, male sterility is often harnessed by using male-sterile lines as female parents to facilitate hybridization, thereby reducing labor and conserving parental resources.

The development of male gametophytes (pollen grains) in anthers involves both meiotic and mitotic division. During meiosis, the diploid microspore mother cells divide to produce a tetrad of haploid microspores. Each microspore subsequently undergoes mitotic division, forming a bicellular with distinct vegetative and generative cells [5, 6]. Lipids and carbohydrates accumulation then lead to pollen grains mature [5]. The pollen wall plays a crucial role for protection of the developing pollen from physical and biological stressors and for critical communication between male and female gametophytes [7]. The mature pollen wall comprises two layers, an outer exine and an inner intine [8]. The intine layer, resembling typical plant cell walls, is primarily composed of cellulose, pectins and cell wall-associated proteins [9]. The exine layer consists of sporopollenin, which is rich in long-chain fatty acids (LCFA), phenylpropanoids and phenolics [10, 11]. Despite its importance, aspects of sporopollenin remain unresolved, including the isolation of its monomer, its chemical properties, and the mechanism of precursors transport from tapetal cells to microspore surfaces for exine formation [12]. The vital role of fatty acids in pollen wall and anther cuticle underscores the importance of lipid metabolism and transport in plant fertility. Pollen wall development and anther cuticle formation share mechanisms in lipid biosynthesis [10, 11, 13]. Genes encoding fatty acid reductase, fatty acyl-CoA synthetase (ACOS), long-chain fatty acid ω-hydroxylase (CYP704B), cytochrome P450 fatty acid hydroxylase (CYP703A), glucose-methanol-choline (GMC) oxidoreductase, and GDSL lipase are shared in these pathways [14]. Exine component transport, particularly lipid transport, is mediated by ATP-binding cassette (ABC) transporters and lipid transfer protein (LTP). In *Arabidopsis*, the *ABCG* genes display functional redundancy in developing anthers and pollen. For instance, the *abcg1/16* double mutant and the *abcg1/16/20* triple mutant anthers release less pollen grains, which are often shriveled [15]. Similarly, the mutation of *OsABCG3*, a homologous gene of *AtABCG16* in rice*,* results in sterile pollen similar to the *abcg1/16* mutant in *Arabidopsis* [16]. *AtABCG26*, *AtABCG9*, *AtABCG31*, and *AtABCG32* are all essential for pollen exine synthesis in *Arabidopsis* [17–21]. The anther cuticle protects plant from biotic stressors and prevents flower ectopic interorgan fusion [22]. Additionally, cuticular wax facilitates pollen hydration and stigma recognition in *Arabidopsi*s. The genes *AtLACS1/4, AtCER1/3/6* participate in the biosynthesis of very long chain fatty acids (VLCFA), influencing exine formation [23–27]. Although pollen hydration in tomato is not dependent on pollen coat lipids, mutation of *SlCER6* impairs floral morphology, exine formation, and fertility [28], underscoring the cuticle’s significance in plant sexual reproduction and flower development.

The pollen wall development and lipid metabolism are tightly regulated by transcriptional factors (TFs). In higher plants, MALE STERILITY 1 (MS1), a plant homodomain (PHD)-finger class TF, is critical for tapetal development and microspore maturation. In *Arabidopsis*, MS1 regulates pollen coat development by influencing LTP expression [29–31]. The bHLH-type TF *Aborted Microspores* (AMS) regulates sporopollenin biosynthesis in *Arabidopsis* by directly activating genes such as *LTPs*, *ABCG26/WBC27*, and *CYPs* [32, 33]. AMS also modulates *MS188* expression, a v-myb avian myeloblastosis viral oncogene homolog (MYB) TF expressed in the tapetum, which directly regulates the expression of a CYTOCHROME P450 gene (*CYP703A2*), *PKSB*, *MALE STERILE2* (*MS2*), and *POLYKETIDE SYNTHASE A* (*PKSA*) [34, 35]. AMS and MS188 may create a feed-forward loop, enhancing the sporopollenin biosynthesis pathway in pollen wall formation [36]. In tomato, SlHB8, a HD-Zip III transcription factor, acts as a negative regulator in tapetum development and pollen wall formation [37]. Variations in *ABORTED MICROSPORES* (*SlAMS*) expression impact pollen viability and pollen morphology in tomato [38]. TFs also regulate pollen development via pathways involving plant hormones [39], glycometabolism [40], and autophagy in tomato [41].

The NAC (NAM, ATAF1/2, and CUC2) TFs, one of the largest plant-specific TF families, regulate diverse plant biological processes [42]. Numerous studies have identified NAC TFs in the regulation of fruit ripening and quality [43]. For example, MaNAC1-MaNAC6 in banana fruits (*Musa acuminata*) regulates fruit ripening through interactions with the ethylene biosynthesis and signal transduction pathway [44]. In strawberry fruits (*Fragaria chiloensis*), FcNAC1 modulates the fruit ripening by regulating pectin metabolisms in response to hormonal signals [45]. The NAC TF BLOOD (BL) in peach fruit (*Prunus persica*) forms a heterodimer with PpNAC1 to promote anthocyanin accumulation during fruit ripening [46]. AdNAC6 and AdNAC7 in kiwifruit (*Actinidia deliciosa*) stimulate the biosynthesis of terpene aroma compounds during fruit ripening [47]. NAC TFs also modulate traits related to fruit senescence, seed development, color change, and flavor release. In tomato, SlNOR and SlNOR-like1 TFs exhibit a high amino acid sequence homology (62.84%) and share multiple target genes that are associated with fruit ripening, seed development, and hormone signaling [43, 48–50]. Their homologs in *Arabidopsis*, *NARS1* (*ANAC056*) and *NARS2* (*ANAC018*), exhibit functional redundancy in regulation of seed development [51], suggesting a possible analogous role in tomato.

In this study, we revealed that SlNOR and SlNOR-like1 possess a redundant function in regulating of pollen development in tomato. We observed that the ovaries of the *nor*/*nor-like1* double mutant fails to undergo proper ovary enlargement and fruit development in each inflorescence due to pollen abortion. The pollen sterility appears to be related to the pollen wall collapse and content leakage. SlNOR and SlNOR-like1 directly regulate the expression of *ABCG* family members and genes coding VLCFA synthases and glycine-rich proteins, which likely contributes to the exine lipid transport, cuticular wax biosynthesis, and sporopollenin deposition. Overall, our findings highlight that SlNOR and SlNOR-like1 redundantly regulate pollen development, providing new insights into transcriptional regulation of flower development.

## Results

### Knockout of *SlNOR* and *SlNOR-like1* leads to pollen abortion

**Figure 1:**
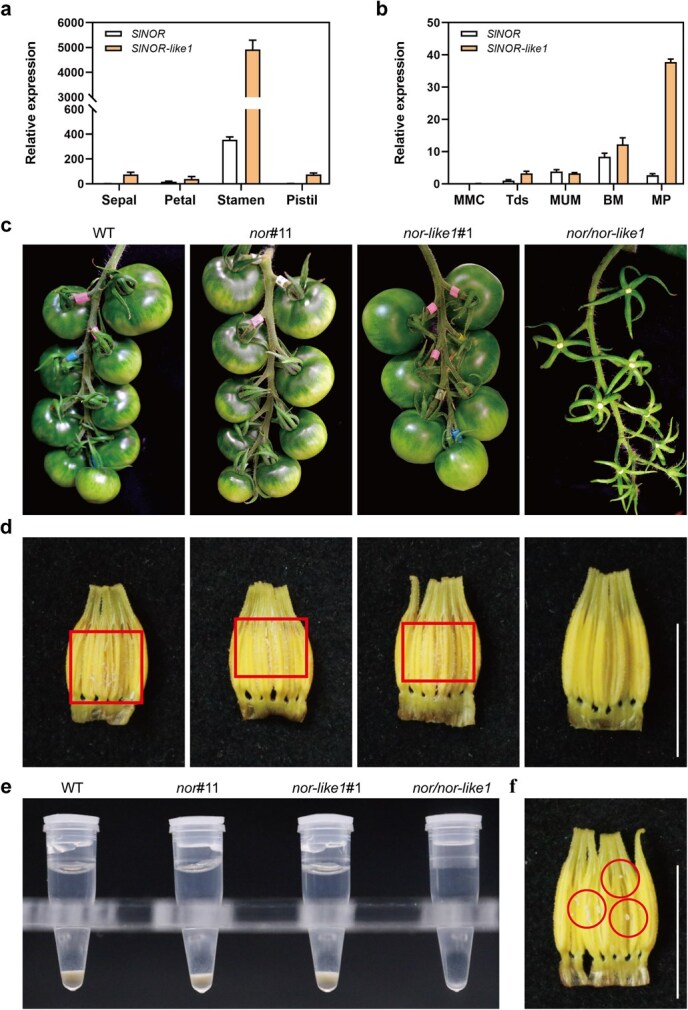
The expression pattern of *SlNOR* and *SlNOR-like1* genes in the WT flower, and the floral phenotype of *nor/nor-like1* compared with WT, *nor*#11, and *nor-like1*#1. a) The relative expression of *SlNOR* and *SlNOR-like1* at different flower organs. (b) The relative expression of *SlNOR* and *SlNOR-like1* in WT stamen at the different developmental stages. **MMC**, microspore mother cell stage; **Tds:** tetrad stage; **MUM:** middle uninucleate microspore stage; **BM:** binucleate microspore stage; **MP:** mature pollen stage. Error bars (a and b) indicate ±SD of three biological replicates. (c) The phenotype of *nor/nor-like1* fruits after pollination compared with WT, *nor*#11*,* and *nor-like1*#1. (d) The pollen grains released on the mature anthers’ inner surface of WT, *nor*#11, *nor-like1*#1*,* and *nor/nor-like1*. Red marked as pollen grains on the inner surface of the anther. (e) The quantity of pollen released from the stamens through artificially assisted vibrations. Five floral pollen grains from WT, *nor*#11, *nor-like1*#1*,* and *nor/nor-like1* were collected into the 200-μl centrifuge tube. (f) Pollen grains in clumps were dissected out from *nor/nor-like1* anthers. Marker indicated pollen grains in clumps. Scale bars: (d and f) 1 cm.

To examine whether *SlNOR* and *SlNOR-like1* regulate the development of tomato flowers, we isolated sepals, petals, stamens, and pistils from blooming flowers of wild-type (WT) tomato plants. We then analyzed the relative expression levels of *SlNOR* and *SlNOR-like1* in these floral organs using quantitative reverse transcription PCR (RT-qPCR). The results showed that both genes were predominately expressed in the stamen, with *SlNOR-like1* showing higher expression than *SlNOR* (Fig. 1a). Further expression analysis revealed that expression of both *SlNOR* and *SlNOR-like1* genes was significantly up-regulated during the binucleate microspore (BM) and mature pollen (MP) stages, suggesting their critical roles in stamen development (Fig. 1b).

**Figure 2:**
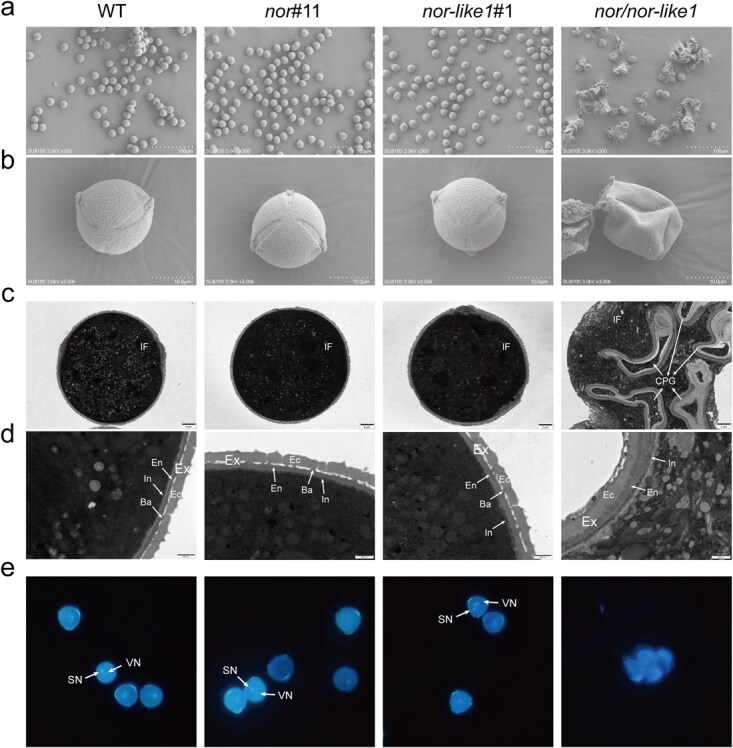
Subcellular features of mature pollens in the *nor/nor-like1* mutant lines compared with WT, *nor*#11 and *nor-like1*#1single mutant lines. (a and b) SEM indicated that the *nor/nor-like1* pollen grains were collapsed and adhesive. There was no obvious difference between WT and single-gene knockout mutants. (c and d) TEM indicated that the structure of the *nor/nor-like1* pollen wall was aberrant. There was no obvious difference between WT and single-gene knockout mutants. **IF:** intracellular fluid; **Ex:** exine; **Se:** sexine; **Ne:** nexine; **Ba:** baculum; **In:** intine; **CPG:** collapsed pollen grain. (e) 4′,6-diamidino-2-phenylindole staining of mature pollen grains. The nuclei were clearly present in the WT and single-gene knockout mutant pollen, but disappeared in the *nor/nor-like1*. **SN:** sperm nucleus; **VN:** vegetative nucleus

To further explore the roles of *SlNOR* and *SlNOR-like1* in tomato flower development, we generated a homozygous *nor/nor-like1* double knockout mutant line by integrating the previously developed the *nor*#11 and *nor-like1*#1knockout lines [48, 49, 52] (Fig. S1). Although the overall plant phenotype of the double knockout mutant lines resembled that of the single mutant lines (Fig. S2a), the *nor/nor-like1* mutant plants displayed abnormal flower morphology, particularly in the stamen, which was elongated by approximately 0.1 mm compared to WT and single mutants (Fig. S2c, d, f). Abnormal stamen of the *nor/nor-like1* failed to pollinate, leading to zero fruit set in the double mutant plants (Fig. 1c). Further examination of mature anthers in the *nor/nor-like1* plants revealed an absence of pollen grains on the inner surface, in stark contrast to WT and single mutants (Fig. 1d and e).

Upon dissecting the *nor/nor-like1* anthers, clumped pollen grains were observed within the anther but failed to be released (Fig. 1f). Additionally, the stamen structure of the *nor/nor-like1* double mutant plants appeared to be stiffer than that of WT, the single mutant *nor*#11 and *nor-like1*#1 lines during dissection (data not shown). This complete abortion in the *nor/nor-like1* plants indicates that SlNOR and SlNOR-like1 play significant roles in regulating pollination, potentially through their impact on stamen development and function.

### Double mutation of *SlNOR* and *SlNOR-like1* causes pollen grain collapse

To investigate cellular-level morphological changes in the stamen and pistil, longitudinal sections of blooming-stage flowers from WT, *nor*#11*, nor-like1*#1, and *nor/nor-like1* plants were examined under an optical microscope. The results showed that the pollen grains of the *nor/nor-like1* mutant plants exhibited reduced numbers, deformities, and adhesion to each other while the stamens, pistils, ovules, and pollen grains in WT, *nor*#11, and *nor-like1*#1 plants all appeared morphologically normal. Notably, despite these abnormalities in the pollen grains, the pistil and ovule structure in the *nor/nor-like1* flowers remained unaffected (Fig. S3).

To further investigate structural changes in the pollen grains, we performed scanning electron microscopy (SEM) and transmission electron microscopy (TEM) analyses. SEM images showed that the pollen grains in the *nor/nor-like1* double mutant had an irregular and collapsed appearance (Fig. 2a and b). TEM analysis revealed that these pollen grains had broken cell walls with intracellular fluid leakage, leading to cellular adhesion (Fig. 2c). In contrast, the pollen walls of WT, *nor*#11, and *nor-like1*#1 single mutant lines displayed two distinct layers, an outer exine and an inner intine, with a clear separation between the sexine and nexine structures. However, in the *nor/nor-like1*lines, the pollen exine lacked supportive gaps provided by the baculum, resulting in a nontypical exine architecture (Fig. 2d). To assess the nuclear integrity of pollen grains, we performed the 4′,6-diamidino-2-phenylindole (DAPI) staining. In WT, *nor*#11 and *nor-like1*#1 lines, mature pollen grains had two nuclei, corresponding to the sperm nucleus and the vegetative cell nucleus. In contrast, the *nor/nor-like1* pollen grains displayed no nucleus signal, indicating that the nuclei were lost along with the intracellular fluid (Fig. 2e). These findings suggest that the disrupted pollen wall structure in the *nor/nor-like1* mutant lines led the collapse of pollen grains, which may be one of causes for pollen abortion.

**Figure 3:**
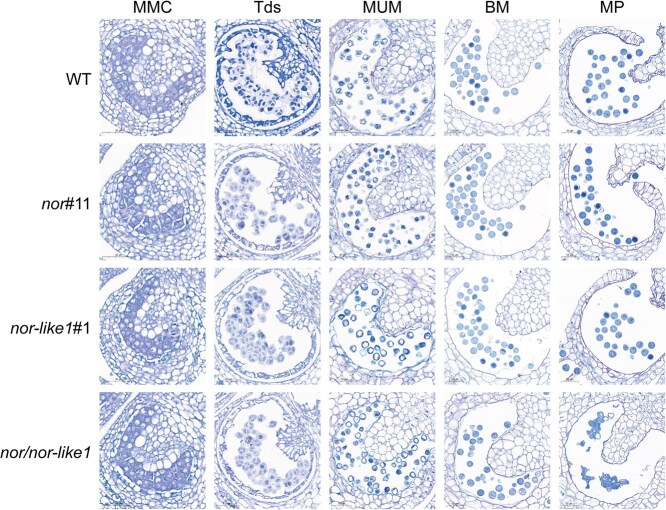
Transverse sections of flower buds at different pollen development stages in *nor/nor-like1*, compared with WT, *nor*#11, and *nor-like1*#1. **MMC:** microspore mother cell stage; **Tds:** tetrad stage; **MUM:** middle uninucleate microspore stage; **BM:** binucleate microspore stage; **MP:** mature pollen stage

Histological analyses showed that while pollen grains in the *nor/nor-like1* mutant plants appear normal in the first four stages, they exhibit severe collapse and adhesion during the MP stage (Fig. 3), which suggests that the defects become evident only in the later stages of pollen maturation. Pollen wall formation is initiated during the early developmental stages, particularly at Tds and MUM, where key biological processes occur. To further understand the effects of *SlNOR* and *SlNOR-like1* on pollen wall development, we performed a subcellular analysis using TEM on pollen at the MUM stage from WT, *nor*#11, *nor-like1*#1, and *nor/nor-like1* plants. TEM revealed that the pollen wall structure in the *nor/nor-like1* at MUM was similar to that of WT, *nor*#11, and *nor-like1*#1(Fig. S4a), with no significant differences in wall thickness observed among these lines (Fig. S4c). However, the *nor/nor-like1* mutant exhibited less component deposition on the exine surface and a reduced gap between sexine and nexine layers compared to WT and single mutants (Fig. S4b). However, these structural differences may not be attributable to abnormalities in the tapetum, as no significant differences in tapetal structure were observed across the different lines (Figs 3 and S5). While these minor structural discrepancies did not result in pollen collapse during the early stages, they likely contributed to the vulnerability of mature pollen in the *nor/nor-like1*mutant line, predisposing it to collapse at MP.

These findings, along with the expression patterns of *SlNOR* and *SlNOR-like1*, suggest that these TFs primarily regulate pollen wall development between the BM and MP stages. We hypothesize that the observed collapse of pollen grains in the *nor/nor-like1*mutant plants, which leads to the fertilization failure, may be due to an abnormal pollen wall structure.

### Low viability of the *nor/nor-like1* pollens severely impairs fertilization

**Figure 4:**
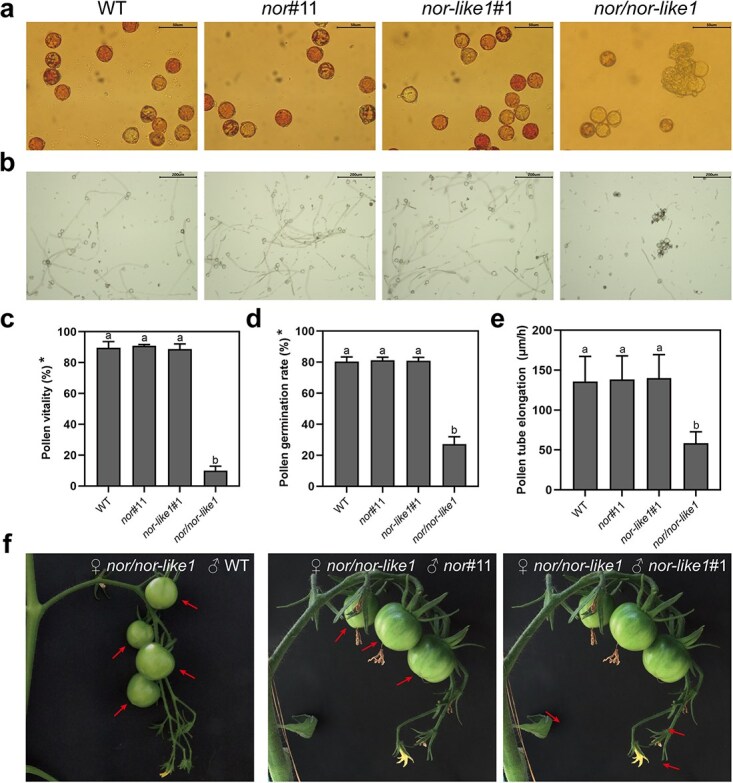
Pollen viability of *nor/nor-like1*, compared with WT, *nor*#11, and *nor-like1*#1. (a and c) The *in vitro* TTC staining assay indicates that pollen viability of *nor/nor-like1* is significantly lower. (b and d) The *in vitro* germination assay reveals that the pollen germination rate of *nor/nor-like1* is significantly lower. (e) The *in vitro* germination assay shows a slower pollen tube elongation of *nor/nor-like1* compared to WT and single mutant lines. Asterisks (c and d) indicate that the statistical rate is significantly higher than the actual situation due to adhering pollen influencing the counting process. Significant differences (*P* < 0.01) are indicated by lowercase letters. (f) The phenotypes from the back-crosses between *nor/nor-like1* and WT, *nor*#11, *nor-like1*#1. The female pistils of *nor/nor-like1* as female parents were successfully pollinated with pollens from WT and single knockout mutants. No observable developmental anomalies were detected in the *nor/nor-like1* fruits. The pollinated flowers were marked with arrows for easy identification

Pollen viability analyses using the triphenyl tetrazolium chloride (TTC) staining indicated that a significant proportion of *nor/nor-like1* pollen grains failed to stain red, which suggests a notably reduced viability compared to WT, *nor*#11 and *nor-like1*#1 (Fig. 4a and c). This reduced viability was further supported by an in vitro germination assay, where the *nor/nor-like1* pollens displayed poor germination capacity (Fig. 4b and d) and slower pollen tube elongation (Fig. 4e). It is worth noting that the sticky nature of *nor/nor-like1* pollen grains affected the microscopic observations, leading to an underestimated pollen count (Fig. S6). While the pollen grains of WT, *nor*#11*,* and *nor-like1*#1 exhibit normal germination rates, the collapsed pollen grains of *nor/nor-like1* exhibited very low viability, resulting in poor germination. To confirm that *nor/nor-like1* pollen grains are unable to complete fertilization, we conducted backcross experiments. After removing the anthers from flowers, pistils from various inflorescences on the same *nor/nor-like1* plant were pollinated with WT, *nor*#11, and *nor-like1*#1 pollens. All cross-back fertilizations were successful, leading to ovary development and fruit formation in the *nor/nor-like1* mutant plants (Fig. 4f). In contrast, other pistils in the same inflorescence without the backcross failed to expand. These findings confirm that, while *nor/nor-like1* pistils retain functional female gametophytes, the *nor/nor-like1* pollen is incapable of achieving fertilization in vivo.

### Double mutation of *SlNOR* and *SlNOR-like1* affects the expression of genes related to pollen wall component metabolism

**Figure 5:**
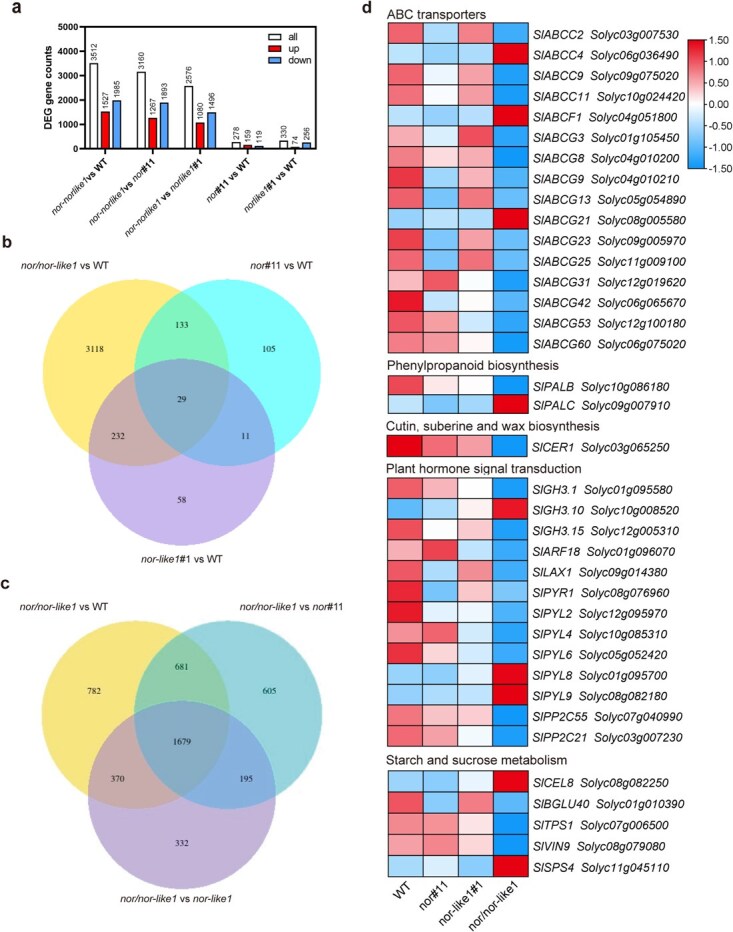
Identification of differentially expressed genes (DEGs) in WT, *nor*#11*, nor-like1*#1, and *nor/nor-like1* stamens before the mature pollen stage by transcriptome analyses. (a) DEG status between *nor/nor-like1* and WT/*nor*#11/*nor-like1*#1, *nor*#11/*nor-like1*#1 and WT; (b and c) Venn diagram and Kyoto Encyclopedia of Genes and Genomes (KEGG) enrichment pathway analysis; (d) The expression level of DEGs related to pollen development in WT, *nor*#11*, nor-like1*#1, and *nor/nor-like1* stamens

To investigate the molecular mechanisms underlying pollen abortion in the *nor/nor-like1* mutant, we performed a transcriptome analysis on stamens collected between the BM and MP stages (Table S6; Fig. S7a and b). RNA-sequencing (RNA-seq) results identified 278, 330, and 3512 differentially expressed genes (DEGs; log₂|fold change| ≥ 1 and adjusted *P*-value ≤0.05) in *nor*#11, *nor-like1*#1, and *nor/nor-like1* stamens, respectively, relative to WT (Figs 5a, b and S7c). When comparing *nor/nor-like1* to the single mutants *nor*#11 and *nor-like1*#1, we identified 3160 and 2576 DEGs, respectively (Figs 5a, c and S7c). Further analyses indicated that there were 1679 common DEGs among the comparisons of *nor/nor-like1* vs WT, *nor*#11 vs WT, and *nor-like1*#1 vs WT (Fig. 5c). Kyoto Encyclopedia of Genes and Genomes (KEGG) enrichment pathway analysis showed that SlNOR and SlNOR-like1 redundantly regulated some genes involved in several key pathways, including ABC transporter, phenylpropanoid biosynthesis, cutin, suberine and wax biosynthesis, hormone signal transduction, and starch and sucrose metabolism pathway (Figs 5d and S8 and Table S7).

Given the collapse phenotype observed in the *nor/nor-like1* pollen grains, we hypothesized that the altered wall composition in the *nor/nor-like1* pollens could be responsible for pollen collapse and abortion. Phenylpropanoids are essential for proper exine formation. Many genes related to the phenylpropanoid biosynthesis pathway were expressed differently in the *nor/nor-like1* stamen when compared to single gene mutants and WT (Fig. S8). For instance, phenylalanine ammonia-lyase (PAL), a key enzyme in the phenylpropanoid pathway, catalyzes the synthesis of p-coumaroyl-CoA [53]. In *Arabidopsis*, the *pal1–1 pal2–1* double mutant was male sterile and unable to produce seeds [54]. The *pal1, pal2, pal3*, and *pal4* quadruple mutants exhibited a more severe phenotype, stunted, and sterile [55]. In the *nor/nor-like1*, *SlPALB* and *SlPALC,* homologous genes of *PAL2* and *PAL1* in *Arabidopsis*, were significantly downregulated (Fig. 5d). Plant ABC transporters, particularly those in the G family, play critical role in various cellular processes, including hormone homeostasis, detoxification, antibiotics resistance, and secondary metabolism [56]. ABC transporters of the G family have been shown to transport precursors of pollen wall components in *Arabidopsis* and *Rice* [15–20, 56, 57]. Some of them are responsible for the transport of sporopollenin precursors, which is essential for pollen to maintain fertility. In the *nor/nor-like1*, the expression of several ABC transporters, especially those in the G family, was markedly down-regulated compared to WT and single mutants. Additionally, multiple genes related to the fatty acid metabolism that is important for pollen wall lipid composition, showed a lower expression in *nor/nor-like1* than those in WT and single gene mutants (Fig. S8, Table S7). These results suggest that both SlNOR and SlNOR-like1 play crucial roles in the regulation of lipid metabolism and transport for pollen wall formation.

Pollen development also relies on hormones and carbohydrates metabolism [40, 58]. In *nor/nor-like1*, several genes associated with starch and sucrose metabolism, including *SlCEL8*, *SlBGLU40*, *SlVIN9,* and *SlTPS1,* exhibited decreased expression (Fig. 5d, Table S7). Additionally, genes involved in auxin signaling, such as auxin influx carrier protein gene (*SlLAX1*), auxin response factor (ARF) genes, and members of Gretchen Hagen 3 (GH3) family, along with abscisic acid (ABA)-related genes encoding protein phosphatase 2C (PP2C) and PYR/PYL/RCAR ABA receptor (PYL)*PYL* family members, showed differential expression, suggesting altered hormone signal transduction pathways in the *nor/nor-like1* mutant plants (Table S7).

Although no significant structural abnormalities were detected in *nor/nor-like1* pollen grains at MUM stage, transcriptome comparisons of WT, *nor*#11, *nor-like1*#1, and *nor/nor-like1* stamens at this stage revealed continued differential enrichment of pathways related to ABC transporters, lipid metabolism, and phenylpropanoid biosynthesis, which are critical for pollen wall development (Fig. S9; Table S8). Notably, *SlABCG8*, *SlABCG9*, and *SlABCG23* were also prominent DEGs at the MUM stage (Table S8), underscoring the role of *SlNOR* and *SlNOR-like1* in the regulation of ABC transporter genes, which warrants further investigation.

### Direct binding of *SlNOR* and *SlNOR-like1* to *SlABCG8/9/23*, *SlCER1*, and *SlGRP92* promoters and activation of gene expression

**Figure 6:**
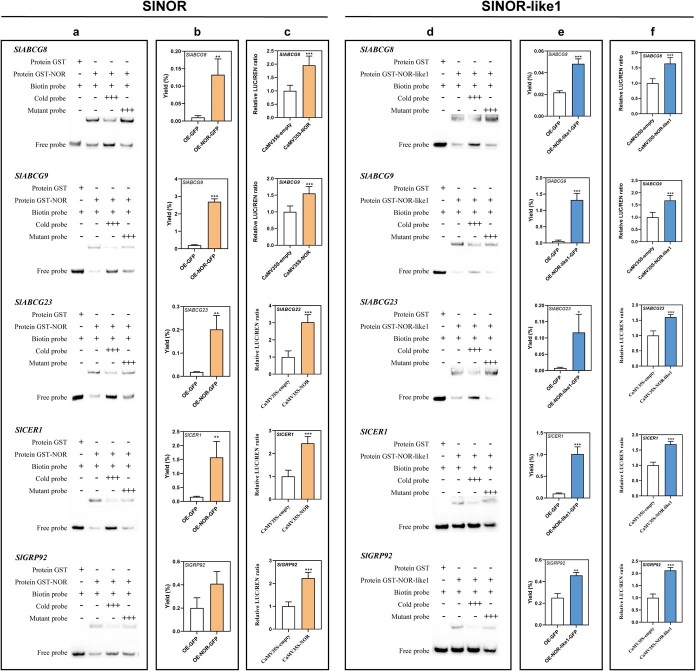
SlNOR and SlNOR-like1 directly bind to *SlABCG8/9/23*, *SlCER1*, and *SlGRP92* promoters and activate their expression. The EMSA shows that SlNOR (a) and SlNOR-like1 (d) directly bind to the target gene promoters containing the NACRS (NAC recognized sequence). The biotin probes undergo a significant shift upon binding with proteins. The dual-luciferase reporter assay (DLR) indicates that SlNOR (b) and SlNOR-like1 (e) interact with promoter regions of five selected genes and activate their expression. ChIP-qPCR analysis confirms the direct binding of SlNOR (c) and SlNOR-like1 (f) to the target gene promoters. The values represent the percentage of DNA fragments in over-expressing (OE)-*GFP* (*NOR*/*NOR-like1* native promoter::GFP) (CK) or over-expressing (OE)-*NOR*/*NOR-like1* (*NOR*/*NOR-like1* native promoter: *NOR/NOR-like1-GFP*) fruit that coimmunoprecipitated with an anti-GFP tag antibody relative to the corresponding input DNA. The error bars indicate ±SD of three biological replicates. Asterisks indicate significant differences determined by Student’s *t*-test (^*^P < 0.05, ^**^P < 0.01, ^***^P < 0.001)

To identify direct downstream target genes that SlNOR and SlNOR-like1 TFs may regulate, we focused on DEGs from KEGG enrichment pathways associated with pollen development. ABCG transporters are critical for lipid transport, including the pollen wall precursors essential for pollen fertility and the movement of pollen wall precursors essential for pollen fertility [15–19, 56]. In *Arabidopsis*, the *abcg1/16* double mutant and *abcg1/16/20* triple mutant exhibit reduced and collapsed pollen grains at maturity [15]. Notably, the mature pollen grain phenotype of the *nor/nor-like1* tomato mutant is analogous to that observed in the *abcg1/16/20 Arabidopsis* mutants. Analysis of the sequence similarity suggests that *SlABCG8/9/23* in tomato corresponds to *AtABCG1/16/20* in *Arabidopsis*. To verify the interaction with SlNOR and SlNOR-like1, we performed an electrophoretic mobility shift assay (EMSA) using a biotin-labeled probe containing the NAC recognition sequence (NACRS). A mobility shift was observed when the labeled probes from *SlABCG8/9/23* promoter regions were incubated with GST-SlNOR or GST-SlNOR-like1 fusion proteins, confirming a direct interaction (Fig. 6a and d). A dual-luciferase reporter (DLR) assay further demonstrated that SlNOR and SlNOR-like1 TFs activate *SlABCG8/9/23* promoter activities in the presence of target motifs (Fig. 6b and e). Moreover, a chromatin immunoprecipitation (ChIP)-qPCR assay confirmed that SlNOR and SlNOR-like1 proteins could bind to *SlABCG8/9/23* promoters in vivo (Fig. 6c and f). In addition, expression levels of *SlABCG8/9/23* were also upregulated in the stamens of tomato plants overexpressing *SlNOR* or *SlNOR-like1* (Fig. S10).

Given the role of cuticular wax biosynthesis in pollen wall integrity and fertility [22–28], we also examined cuticular wax synthesis genes within the DEGs. Specifically, *SlCER1*, the tomato homolog of *AtCER1* in *Arabidopsis*, is involved in the biosynthesis of very long-chain fatty acids (VLCFAs) [27]. Evidence suggests that glycine-rich proteins are essential for the exine formation and pollen maturation in tomato [59]. We also identified a DEG encoding a glycine-rich protein (*SlGRP92*) in the *nor/nor-like1* transcriptome at the mature pollen stage. EMSA, DLR, and ChIP-qPCR assays confirmed that SlNOR and SlNOR-like1 directly bind to the promoters of both *SlCER1* and *SlGRP92*, activating their expression (Fig. 6). Consistently, *SlCER1* and *SlGRP92* expression levels increased in the stamens of *SlNOR* or *SlNOR-like1* overexpressing plants (Fig. S10). These results suggest that SlNOR and SlNOR-like1 regulate pollen wall formation and fertility by modulating the expression of these essential downstream genes.

## Discussion

### SlNOR and SlNOR-like1 regulate pollen viability by affecting pollen wall integrity at the mature pollen stage

SlNOR and SlNOR-like1 are widely recognized for their pivotal roles in regulating fruit ripening in tomato [43, 48, 49, 60]. Although high expression levels of these genes have been observed in the floral organs [48, 49], their roles in flower development were previously overlooked due to the lack of visible phenotypic changes in the floral organs of *SlNOR*-knockout or *SlNOR-like1*-single knockout mutants. SlNOR and SlNOR-like1 share the highest amino acid sequence homology in tomato [61], with their homologous genes in *Arabidopsis* exhibiting functional redundancy in seed development [51]. We hypothesize that this redundancy likely extends to SlNOR and SlNOR-like1 in tomato. By integrating the *SlNOR*-knockout mutant with the *SlNOR-like1*-knockout mutant, we successfully generated a double knockout mutant (*nor/nor-like1*) (Fig. S1). Our findings indicate that the ovaries in the *nor/nor-like1* inflorescences were incapable to develop into a normal fruit (Fig. 1c), which supports the assumption. Most importantly, this study demonstrates that SlNOR and SlNOR-like1 are essential for pollen development by regulating pollen wall formation. The mutant’s pollen grains, which could not be released from the stamens even with artificial vibrations (Fig. 1d), exhibited collapsed, sticky, and defective characteristics (Fig. 2). Furthermore, the *nor/nor-like1* pollens displayed a low reproductive activity and poor germination rates (Fig. 4a-e), while the female fertility remained intact (Fig. S3). Histological analysis showed that *nor/nor-like1* pollen grains experienced wall rupture and collapse specifically at the mature pollen stage (Fig. 3). Information on the transcriptional regulation of pollen wall formation is scarce in tomato. Pollen wall development requires the accumulation of specific chemical components, notably sporopollenin, which begins at the tetrad stage [5, 10, 12]. Defects in the pollen wall structure compromise pollen stability, often resulting in collapse before pollen maturation [10, 62]. In the *nor/nor-like1* double knockout mutants, subtle structural abnormalities in the exine were observed without significant impact on pollen stability at early stages. Nevertheless, these defects predisposed the mature pollen to collapse (Figs S4 and 3). This delayed collapse may be attributed to the relatively low expression levels of *SlNOR* and *SlNOR-like1* at early developmental stages, limiting their effect on pollen wall formation until the later stages. Interestingly, the pollen shrinkage observed in the *SlMYB33*-RNAi line in tomato at the mature pollen stage has been linked to the cytoplasm degradation [40].

### ABCG lipid transporters as a pathway for SlNOR and SlNOR-like1 in regulating pollen wall synthesis

**Figure 7:**
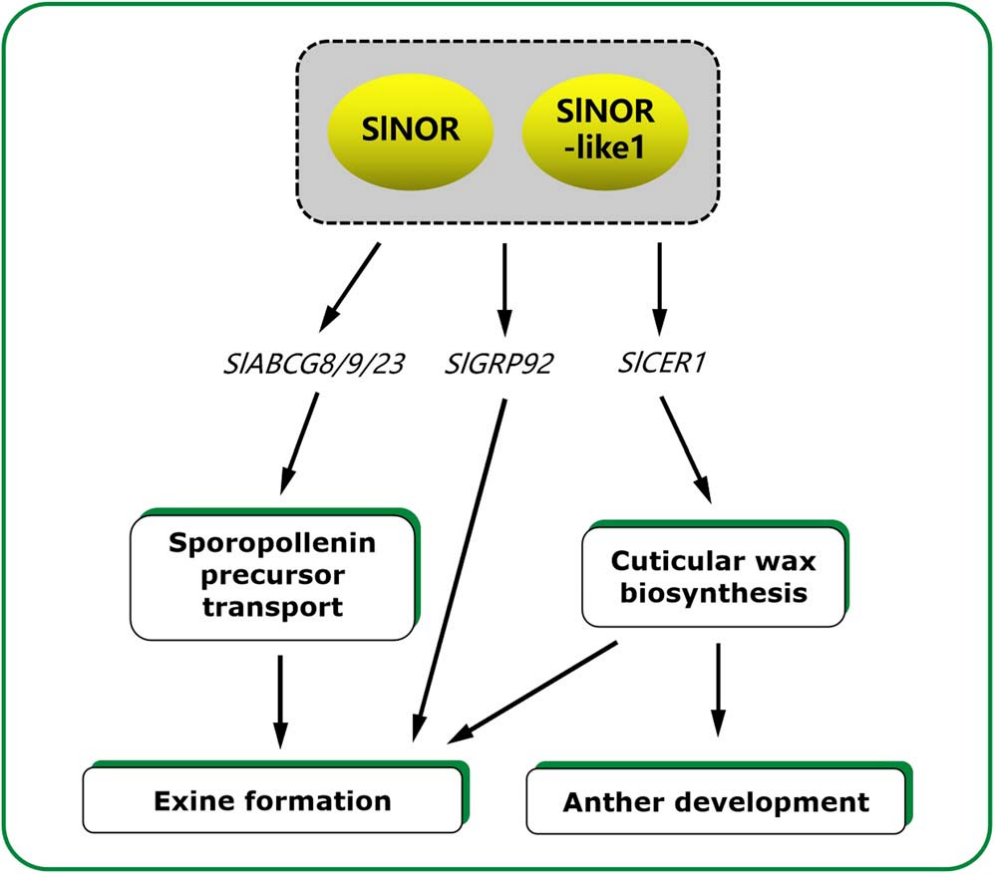
A working model for the SlNOR and SlNOR-like1 mediated regulation of tomato pollen development. In detail, SlNOR and SlNOR-like1 positively regulate the ABCG transporter genes *SlABCG8/9/23* for transporting sporopollenin precursor, the *SlGRP92* gene for pollen exine formation, and the *SlCER1* gene for the cuticular wax biosynthesis for pollen exine composition and stamen development. *SlNOR* and *SlNOR-like1* double knockout mutant (*nor/nor-like1*) exhibit the pollen abortion resulted from the pollen wall collapse and hindered pollen transmission. Positive effects are indicated by arrows

ABC transporters are a diverse class of proteins that actively transport a variety of molecules, including organic acids, metal ions, phytohormones, and secondary metabolites [63]. The exine layer of pollen walls is composed of sporopollenin, a durable biopolymer essential for pollen wall integrity [10, 14]. Sporopollenin precursors are synthesized in the tapetum’s endoplasmic reticulum and transported to the pollen surface via ABC transporters, which play a critical role in pollen wall formation [14]. In particular, these transporters have been implicated in the transport of sporopollenin precursors necessary for exine formation [11, 17–20]. In *Arabidopsis*, pollens from *abcg1/16/20* triple mutant collapse at maturity as the anthers dry and release pollen [15]. Similarly, the loss of function of *OsABCG3*, a homologous gene of *AtABCG16* in rice, results abnormal tapetum degradation and production of shriveled pollen lacking the nexine II and intine layers [16]. The collapsed and adhesive pollen grains observed in the *nor/nor-like1* tomato mutant closely resemble the phenotype of the *abcg1/16/20* mutant in *Arabidopsis* (Fig. 2a and b). Our study reveals that *SlABCG8/9/23*, which are homologous genes of *AtABCG1/16/20* in *Arabidopsis*, are significantly downregulated in the *nor/nor-like1* mutant. EMSA, DLR, and ChIP-qPCR analyses confirm that SlNOR and SlNOR-like1 directly bind to the *SlABCG8/9/23* promoters and activate their expression (Fig. 6). These findings suggest that these homologous genes may share a conserved function across species, with SlNOR and SlNOR-like1 potentially playing a broad regulatory role in ABCG transporter activity (Fig. 5d and Table S3). Given that ABCG transporters are vital for lipid transport during pollen development, SlNOR, and SlNOR-like1 may regulate pollen wall synthesis by modulating the expression of *SlABCG8/9/23* (Fig. 7). However, further studies are needed to identify the specific types of lipids transported by these transporters in tomato stamens.

### Cuticular wax plays an important role in maintaining tomato pollen fertility

The cuticle is a protective layer that separates the outer epidermis cell wall from the surrounding environment in terrestrial plants serving as barrier against various biotic and abiotic stresses, such as drought, UV radiation, temperature fluctuations, pests, and pathogens [64]. The cuticle is mainly made up of cutin and wax. Cuticular wax contains a complex mixture of VLCFAs, triterpenoids, and minor secondary metabolites [65]. In *Arabidopsis*, cuticular waxes are essential for successful pistil–pollen interactions and pollen hydration on the stigma, and for preventing unintended fusion between floral organs [25, 66, 67]. Mutations in *Arabidopsis* CER genes (*cer1, cer2, cer3,* and *cer6*), which are crucial for VLCFA and lipid-rich pollen coat biosynthesis, disrupt pollen hydration, and recognition processes, resulting in male sterility [25, 27, 66]. Long-chain acyl-CoA synthetases (LACSs), specifically *LACS1* and *LACS4*, provide VLCFA-CoAs for wax biosynthesis. The *lacs1/lacs4* double mutant in *Arabidopsis* produces conditionally sterile pollen, which regains fertility under high humidity [23]. Research on the cuticle has primarily focused on *Arabidopsis*, which has a relatively thin cuticle with distinct cutin composition compared to other plants. In tomato, however, pollen hydration does not depend on pollen coat lipids, indicating that cuticular wax may have a different role in sexual reproduction [28]. For example, *SlCER6* in tomato is essential for regulating the timely degradation of tapetum, thereby supporting successful microgametogenesis. Mutations in *SlCER6* lead to impaired pollen dispersal due to flower organ fusion [28]. In other species, such as cotton, the *GhGPAT12/25*, encoding glycerol-3-phosphate acyltransferases plays a pivotal role in synthesizing the glycerol monomers for anther cuticle formation and pollen exine development [68]. In rice, *OsCER1* and *OsWDA1* are involved in VLCFAs biosynthesis, influencing plastid and tapetum development [26, 69]. Our findings suggest that the SlNOR/SlNOR-like1 regulate the *SlCER1* expression, and play a unique role in pollen wall development and stamen morphology in tomato (Fig. 7). This regulatory pathway could be essential for maintaining pollen wall integrity and ensuring pollen fertility, particularly by influencing cuticular wax synthesis.

### The function of *SlNOR* and *SlNOR-like1* in regulating stamen development mirrors their role in tomato fruit

Our study demonstrates that the pollen abortion in the *nor/nor-like1* double knockout mutant is resulted from the collapse of the pollen exine, which likely directly contributes to the absence of fruit formation in these plants. Notably, we observed a lack of viable pollen grains on the inner surface of mature anthers in *nor/nor-like1*, while the anthers in WT and single mutants cracked open, successfully releasing pollen grains (Fig. 1d). In contrast, aborted pollen in *nor/nor-like1* remained trapped within the anthers, possibly due to their stiffer stamen structure (Fig. 1f). Although we did not quantify stamen firmness due to the small size of these structures, previous studies have shown that the loss of either *SlNOR* or *SlNOR-like1* genes results increased firmness in tomato fruit [48, 49]. This suggests that SlNOR and SlNOR-like1 may regulate tissue firmness similarly in both stamen and fruit. Additionally, we observed elongation of the stamen in *nor/nor-like1* plants (Fig. S2b and c), suggesting that *SlNOR* and *SlNOR-like1* may influence stamen size through mechanisms like those that control fruit growth. Differentially expressed genes (DEGs) between *nor/nor-like1* and WT, *nor*#11, or *nor-like1*#1 was enriched in the ‘flavonoid biosynthesis’ pathway included in the metabolites of tomato fruit [48, 49]. This genetic difference may contribute to the lighter yellow color observed in the *nor/nor-like1* stamen. In conclusion, the functions of *SlNOR* and *SlNOR-like1* appear to be conserved across different tissues. These genes regulate similar pathways involved in tissue firmness, size, and color in both tomato stamen and fruit, highlighting their broad regulatory roles within plant growth and development in tomato.

### Redundancy as a barrier for exploring gene function

Functional redundancy among gene families is a widespread phenomenon. Codon degeneracy increases the similarity of amino acid composition in proteins, which can, in turn, lead to similar protein function. This redundancy may serve as an evolutionary strategy to enhance the stability of biological traits. TFs, which act as regulatory ‘switches’ for gene expression, often display extensive functional redundancy [50, 70–73]. In tomato, SlNOR and SlNOR-like1 are critical for fruit ripening, sharing a high amino acid homology and regulating multiple target genes [50]. Their homologs in *Arabidopsis* exhibit functional redundancy in seed development [51]. Our study reveals a similar redundancy in *SlNOR* and *SlNOR-like1* for stamen and pollen wall development. The double mutant *nor/nor-like1* displayed pollen abortion and male sterility, a phenotype missing in the single-gene mutants. Investigating functional redundancy provides deeper insights into molecular evolutionary processes of life. In the practical sense, our findings of the male sterility in the *SlNOR* and *SlNOR-like1* double knockout mutant may provide an alternative approach for generating new hybrid varieties in the future for tomato molecular breeding.

## Materials and methods

### Plant materials and growth conditions

*SlNOR* and *SlNOR-like1* knockout mutant (*nor*/*nor-like1*) were generated through a cross between *SlNOR*-knockout#11 (*nor*#11*)* and *SlNOR-like1*-knockout#1 (*nor-like1*#1*)* plants. To achieve this, *nor*#11 plants, as female parent following emasculation, were pollinated with pollen grains from *nor-like1*#1 plant and subsequently bagged. After harvesting the seeds and propagation, DNA was extracted from the leaves and used as template for amplifying the target sites. The PCR products were sequenced to identify double homozygous mutants (*nor/nor-like1*). WT tomato (*Solanum lycopersicum*) cultivars ‘Ailsa Craig’ and transgenic lines, including *nor*#11*, nor-like1*#1, and *nor/nor-like1* were grown in a greenhouse under a 16-h-light (25°C) and 8-h-dark (20°C) cycle. The *nor* and *nor-like1* single knockout plants were previously described in the earlier studies [48, 49].

### Phenotypic analysis

Inflorescences, stamens, pistils, and fruits were observed and compared among WT, *nor*#11*, nor-like1*#1, and *nor/nor-like1*. A vernier caliper was used to measure the length of pistils as well as the stamens’ length and transverse diameter, from WT, *nor*#11*, nor-like1*#1, and *nor/nor-like1*. Each value represented the means of 20 biological replicates. To determine the fruit set rate, 100 flowers were sampled from 20 plants with similar inflorescences and growth heights. The percentage was calculated as the mean of three biological replicates. For pollen production comparisons, pollens from five flowers of WT, *nor*#11*, nor-like1*#1, and *nor/nor-like1* were collected using artificial aided vibration and placed into a 200-μl centrifuge tube.

### Pollen viability assay

Pollen viability was assessed using the TTC method [37]. Pollen grains from WT, *nor*#11*, nor-like1*#1, and *nor/nor-like1* were collected and incubated in TTC stain at 37°C for 20 min. Active pollen grains were stained red. Pollen viability was calculated by counting more than 100 pollen grains in a microscope field with three replicates. Due to the adhesion of *nor/nor-like1* pollen grains, only dispersed pollen grains were counted, leading to a significantly lower viability rate compared to the statistical average.

Fragkostefanakis’ approach [74] was employed to modify the pollen germination solution [20 mM MES, 1 mM KCl, 3 mM Ca(NO_3_)_2_, 0.8 mM MgCl_2_, 1.6 mM H_3_BO_3_, 2.5% (w/v) Suc, and 24% (w/v) PEG4000] [75]. Pollen staining and deformation were evaluated under an optical microscope. The pollen germination rate was determined by counting over 100 pollen grains per microscope field, with five replicates. Pollen tube elongation was measured by recording the lengths of 40 pollen tubes at 3 h after germinating in the solution.

### Cytological characterization of stamens and pistils

Stamens were collected from WT, *nor*#11*, nor-like1*#1, and *nor/nor-like1* plants at the different developmental stages of microspore mother cell, tetrad, middle uninucleate microspore, binucleate microspore, and mature pollen stages, along with pistils at the mature pollen stage. The samples were fixed in 4% paraformaldehyde at room temperature for 24–36 h. After paraffin embedding and sectioning, stamen and pistil cell characteristics were analyzed under an optical microscope. Mature pollen grains from several tomato lines were stained with .01 mg/ml 4′, 6-diamidino-2-phenylindole (DAPI), and their nuclear state was observed using a fluorescence microscope.

### Electron microscopy of pollen phenotype

Mature pollen grains from various tomato lines were collected and fixed in 2.5% glutaraldehyde at 4°C for 2–4 h. Samples were subsequently dehydrated, dried, and coated with gold for scanning electron microscopy (SEM) using a Hitachi SU8100 scanning electron microscope. Following dehydration and embedding, 80-nm ultrathin slices were prepared for transmission electron microscopy (TEM). Samples were dehydrated, embedded, and sectioned into 80-nm ultrathin slices. These slices were stained with a uranyl acetate substitute and lead citrate before being observed with a Hitachi HT7800 transmission electron microscope.

### Quantitative reverse transcription PCR

Total RNA was extracted from the tomato stamen, pistil, sepal, and petal of various developmental stages using protocols provided by the RNA extraction kit (Omega, USA). cDNA was synthesized using TransScript One-Step gDNA Removal and cDNA Synthesis SuperMix (Vazyme, China). qRT-PCR was performed using SYBR Green PCR Master Mix (Vazyme, China) with a CFX96 Real-Time PCR System (Bio-Rad, USA). The tomato *Actin* gene (Solyc03g078400) was used as the internal control. Relative gene expression levels were calculated using the 2^-△△CT^ method, with three biological replicates [75]. All primers used for qRT-PCR are listed in Table S2.

### Transcriptome profiling

Stamens from WT, *nor*#11, *nor-like1*#1, and *nor/nor-like1* prior to the mature pollen stage were collected and immediately frozen in liquid nitrogen for RNA extraction and sequencing. mRNA enrichment, RNA-seq library construction, and sequencing were performed by Novogene (Beijing, China).

### Back-cross analysis

Two or three pistils from an inflorescence of *nor/nor-like1* plants were selected as female parents and pollinated with pollen grains from WT, *nor*#11*,* and *nor-like1*#1 plants, followed by bagging. The resulting fruit phenotypes were observed and compared with those of other pistils within the same inflorescence without the artificial pollination.

### EMSA

The pGEX-*GST*-*NOR* vector was constructed using a truncated sequence of *NOR* containing the NAC domain, while the pGEX-*GST*-*NOR-like1* vector was generated using the full-length CDS of *NOR-like1*. These vectors were introduced into *Escherichia coli* strain BM Rosetta (DE3) to express GST-NOR and GST-NOR-like1 fusion protein. Protein purification was carried out using A protein purification kit from Beyotime (China). Putative target gene promoter fragments were synthesized as biotin-labelled oligonucleotides, with the promoter fragments containing the NACRS (NAC recognized sequence) [TA][TG][AGC]CGT[GA][TA] used for the assay. The sequences for these fragments are provided in Table S3.

### DLR

The 2-kb promoter regions of *SlABCG8*, *SlABCG9*, *SlABCG23*, *SlCER1*, and *SlGRP92* were cloned into the pGreenII0800-LUC double-reporter vector, while the coding sequences of the *NOR* and *NOR-like1* were cloned into the pCambia1300 vector as effector. These recombinant vectors were separately introduced into *Agrobacterium tumefaciens* strain GV3101. *A. tumefaciens* containing constructed effector and reporter plasmids were co-infiltrated into tobacco leaves. The LUC to REN ratio was subsequently measured using the DLR kit (LABLEAD, China) with six replicates. The primers are listed in Table S4.

### ChIP-qPCR analysis

The ChIP assay was performed as described previously [48, 49] with minor modifications. Briefly, transgenic fruits expressing *NOR* native promoter::GFP, *NOR* native promoter::*NOR*-GFP, *NOR-like1* native promoter::GFP, and *NOR-like1* native promoter::*NOR-like1*-GFP were harvested at the breaker stage. Pericarp tissue was sliced and submerged in 1% (v/v) formaldehyde to cross-link genomic DNA and protein. Nuclei were extracted following cross-linking, and chromatin was fragmented by sonication. The resulting products were immunoprecipitated using an anti-GFP antibody (LABLEAD, China). ChIP DNA, extracted after reverse cross-linking and proteinase K treatment, was used as the template for ChIP-qPCR. Primer sequences for qPCR are listed in the Table S5.

### Statistical analysis

Significance analysis of corresponding experimental data was conducted using IBM SPSS statistics 27 software. Pairwise comparison was computed using Student’s *t*-test (^*^P < 0.05, ^**^P < 0.01, and ^***^P < 0.001), while multiple comparisons were subjected to ANOVA using Duncan test, statistically significant differences were indicated by diverse lowercase.

#### Accession numbers

The accession numbers used in this study are as follows: *SlNOR*, Solyc10g006880; *SlNOR-like1*, Solyc07g063420; *SlABCG8*, Solyc04g010200; *SlABCG9*, Solyc04g010210; *SlABCG23*, Solyc09g005970; *SlCER1*, Solyc03g065250; *SlGRP92*, and Solyc02g032910.

## Supplementary Material

Web_Material_uhaf003

## Data Availability

All relevant data can be found within the article and its supporting materials.
