# Supplementary material for: Functional redundancy of transcription factors SlNOR and SlNOR-like1 is required for pollen development in tomato
Source: Hortic Res. 2025 Jan 6;12(4):uhaf003. doi: 10.1093/hr/uhaf003 (PMC11896966; doi:10.1093/hr/uhaf003)
Supplement: Web_Material_uhaf003 [file web_material_uhaf003.zip › Supplementary file-HR.docx]

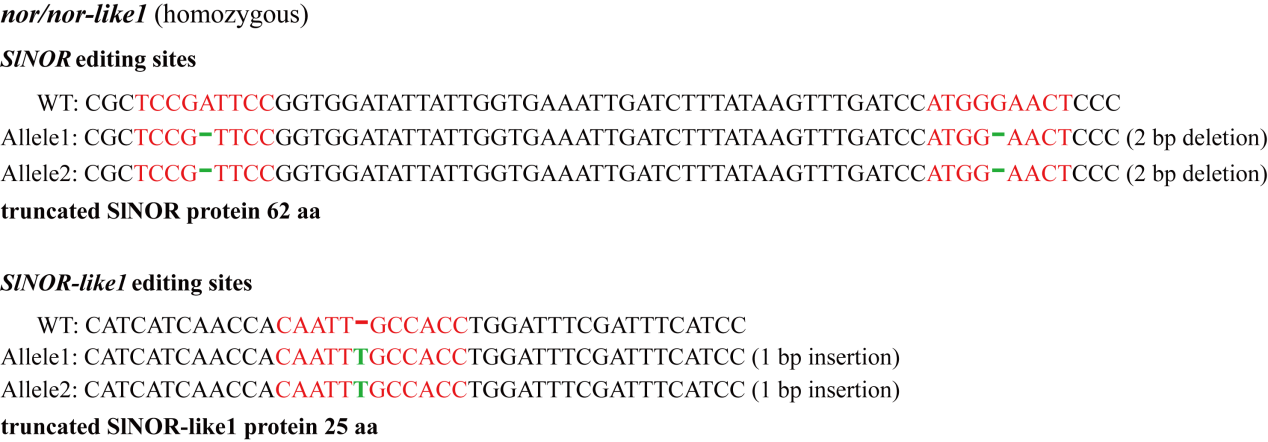


Supplementary Figure S1. Gene editing analysis of *nor/nor-like1* mutant. Red letters indicate the target sites, green letters represent edited site and editing type.


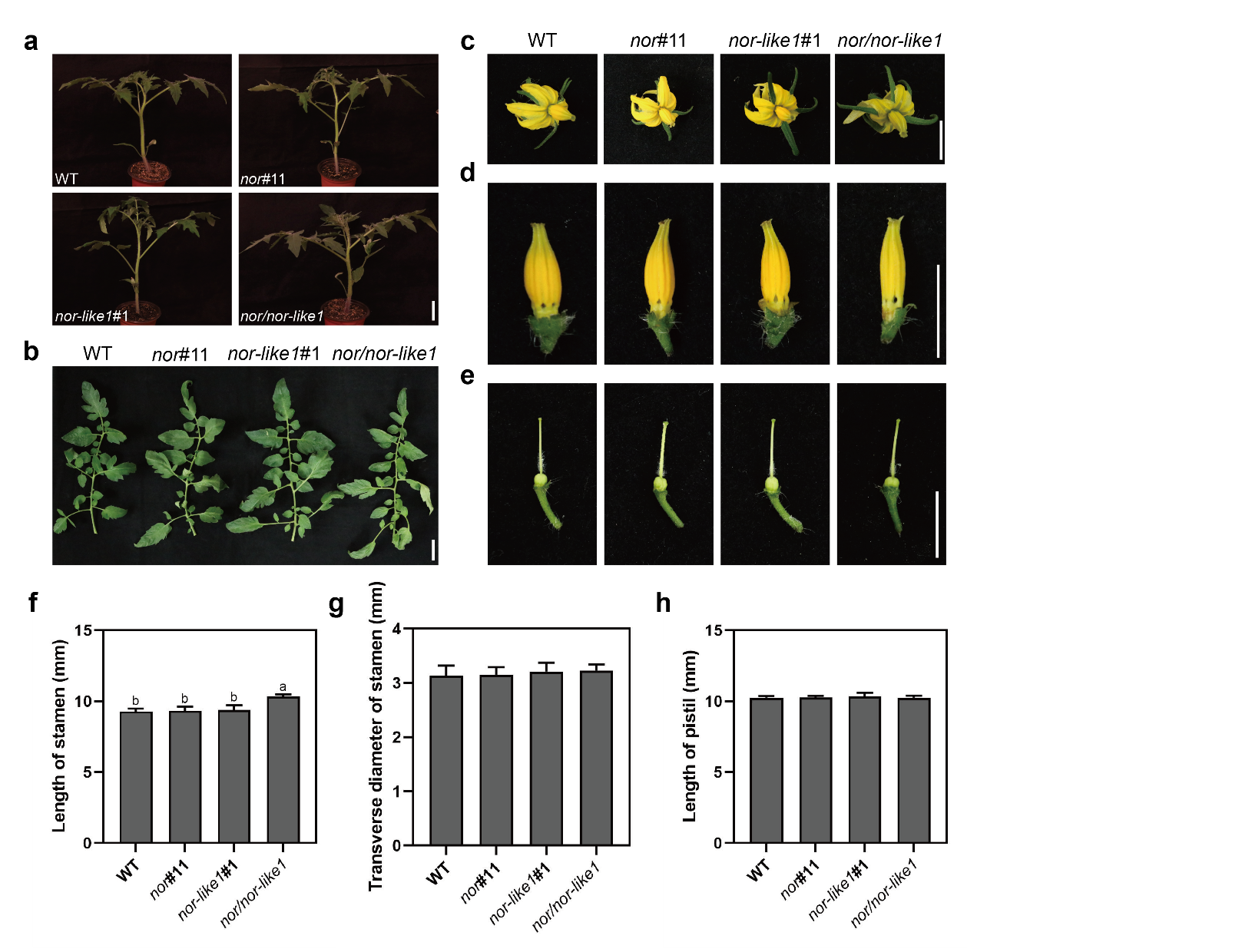


Supplementary Figure S2. The plant growth and flower phenotype of *nor/nor-like1* compared with WT, *nor*#11 and *nor-like1*#1.

a, b) The development of *nor*#11, *nor-like1*#1 and *nor/nor-like1* plants were not affected compared to WT plant. Scale bars: 5 cm. The flower (c), stamen (d), and pistil (e) phenotype of *nor/nor-like1* compared with WT, *nor*#11 and *nor-like1*#1. Scale bars: 1 cm. The length (f) and transverse diameter (g) of stamens, and the length of pistils of *nor/nor-like1* compared with WT, *nor*#11 and *nor-like1*#1. Error bars (a, b) indicate ±SD of 20 biological replicates. Significant differences (P<0.01) are indicated by lowercase letters.


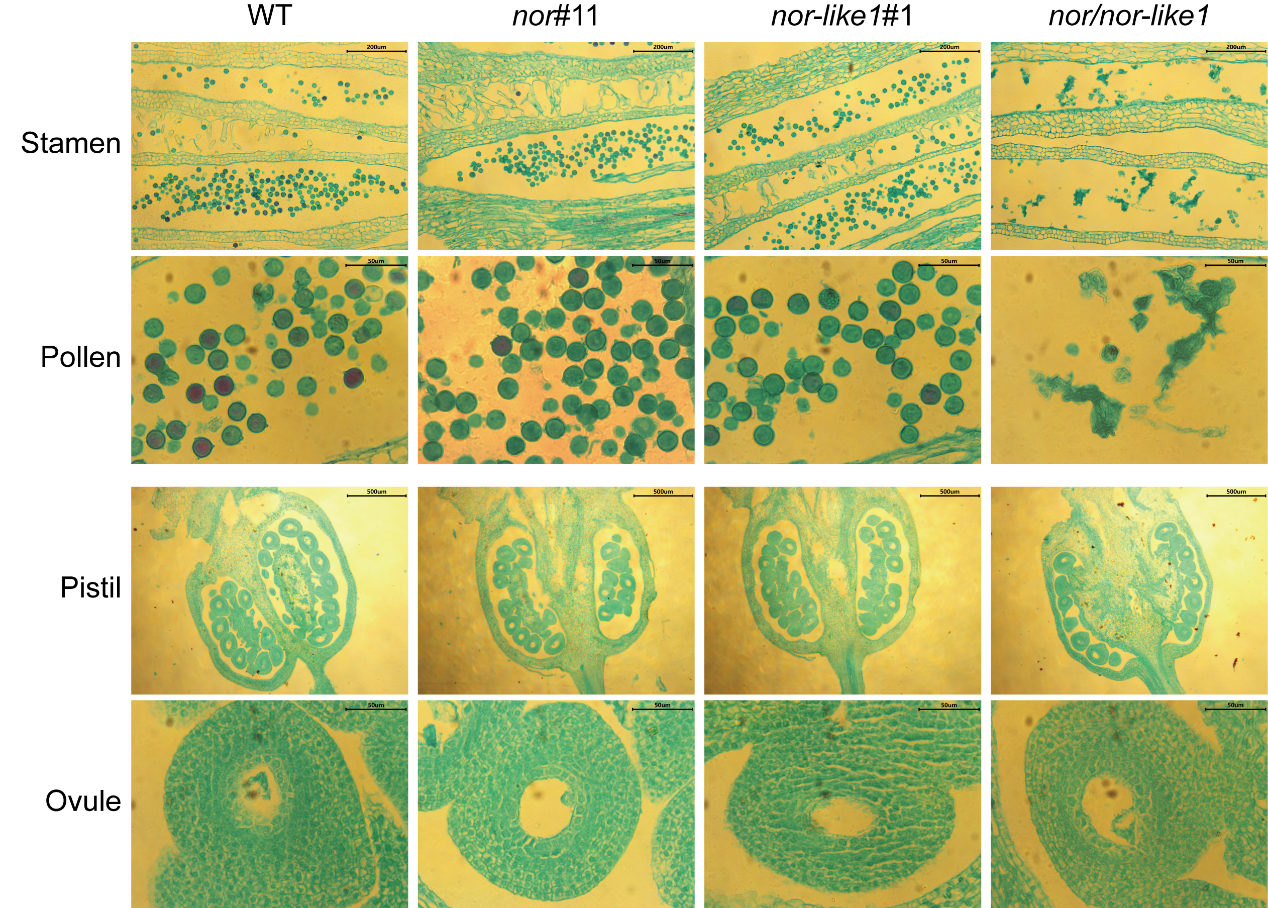


Supplementary Figure S3. The cell morphological structure of *nor/nor-like1* stamen, pollen, pistil and ovule under microscope, compared with WT, *nor*#11 and *nor-like1*#1.


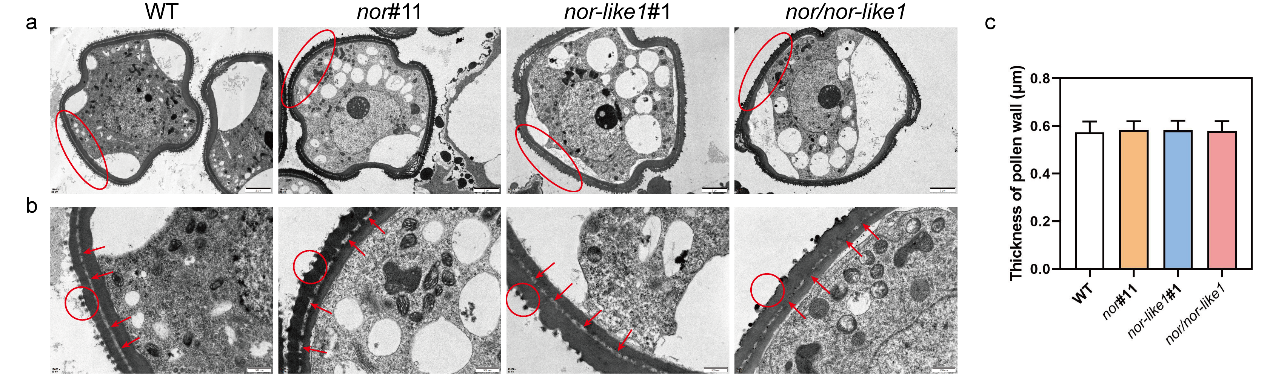


Supplementary Figure S4. Subcellular features of pollen at MUM in *nor/nor-like1* compared with WT, *nor*#11 and *nor-like1*#1.

a) MUM pollen features of *nor/nor-like1* under TEM compared with WT, *nor*#11 and *nor-like1*#1. Red marked as component deposition of pollen wall*.* Scale bars: 2 μm. b) MUM pollen wall features of *nor/nor-like1* under TEM compared with WT, *nor*#11 and *nor-like1*#1. Red circles marked as the component deposition of pollen wall. Red arrows marked as the structural difference of pollen wall between *nor/nor-like1* compared with WT, *nor*#11 and *nor-like1*#1. Scale bars: 0.5 μm. c) The thickness of pollen wall in WT, *nor#11*, *nor-like1#1* and *nor/nor-like1.*


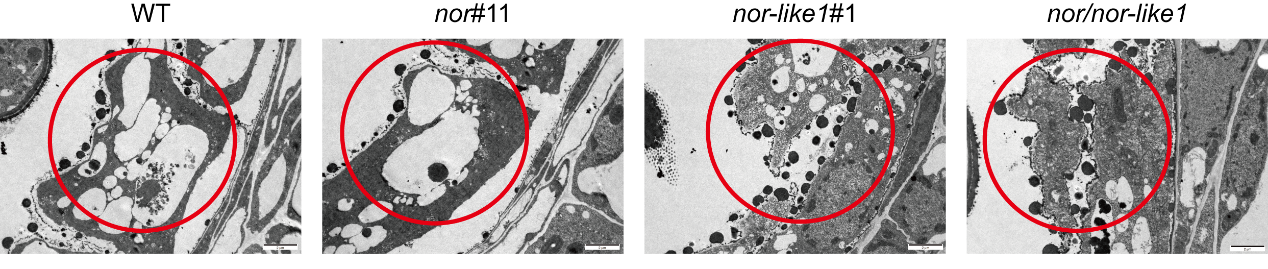


Supplementary Figure S5. Subcellular features of tapetum at MUM in *nor/nor-like1* compared with WT, *nor*#11 and *nor-like1*#1. Red marked as tapetum*.* Scale bars: 2 μm.


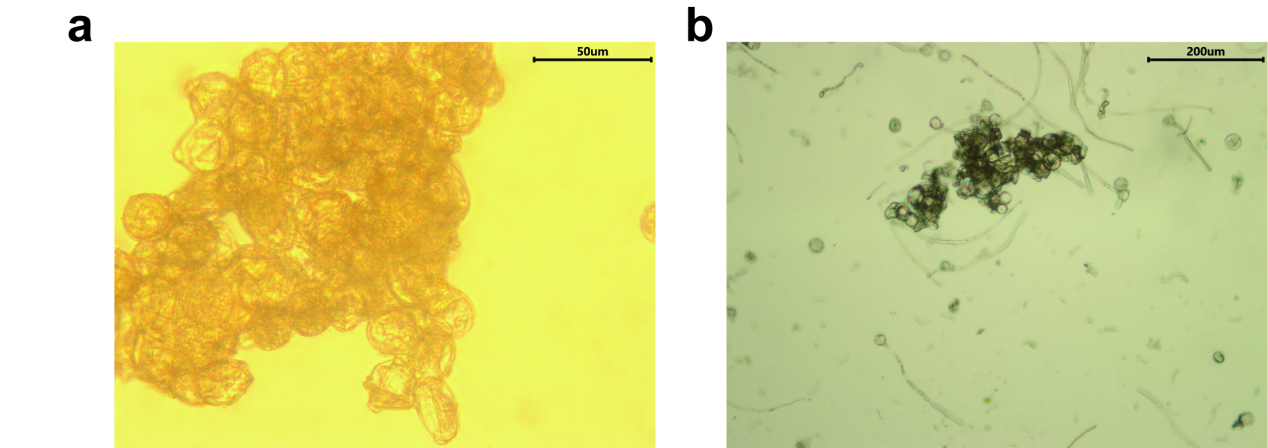


Supplementary Figure S6. The adhesive pollen grains of *nor/nor-like1* in pollen staining assay (a) and pollen vitro germination assay (b).


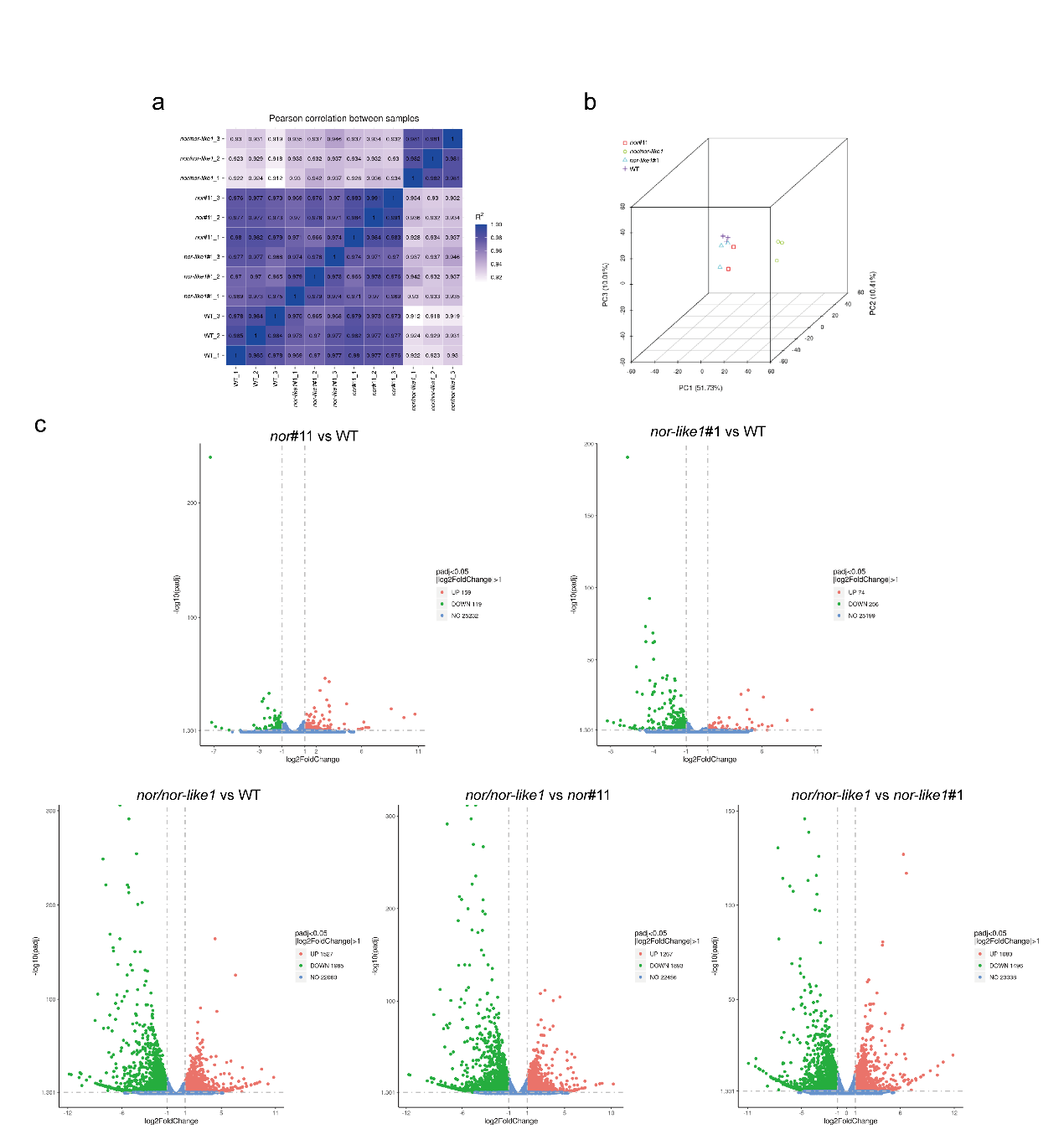


Supplementary Figure S7. RNA-Seq data quality analysis and RNA-Seq data visualized by volcano plots.

a) Pearson correlation analysis of RNA-seq. R^2^ means the square of the pearson correlation coefficient. b) Primary component analyze (PCA) of FPKM in WT, *nor*#11*, nor-like1*#1, and *nor/nor-like1*. The X axis represents the first principal component, the Z axis represents the second principal component, and the Y axis represents the second principal component. c) RNA-seq data visualized by volcano plots. Each point represents a DEG. Red points represent upregulated genes, and green points represent downregulated genes. | Log2 (fold change) |= 1 and p-value = 0.05 are marked with purple lines.


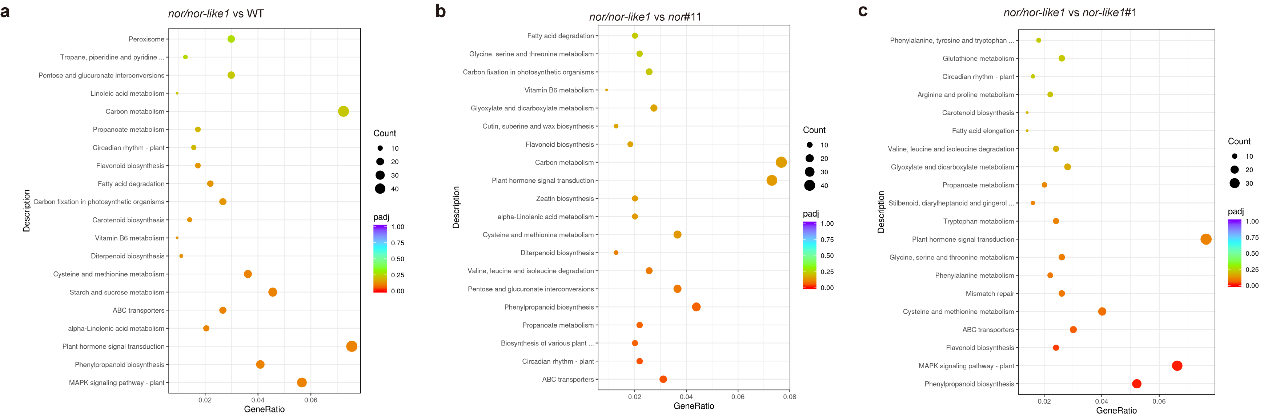


Supplementary Figure S8. Kyoto Encyclopedia of Genes and Genomes (KEGG) enrichment pathway analysis between *nor/nor-like1* stamens and WT/*nor*#11/*nor-like1*#1 stamens before MP stage.


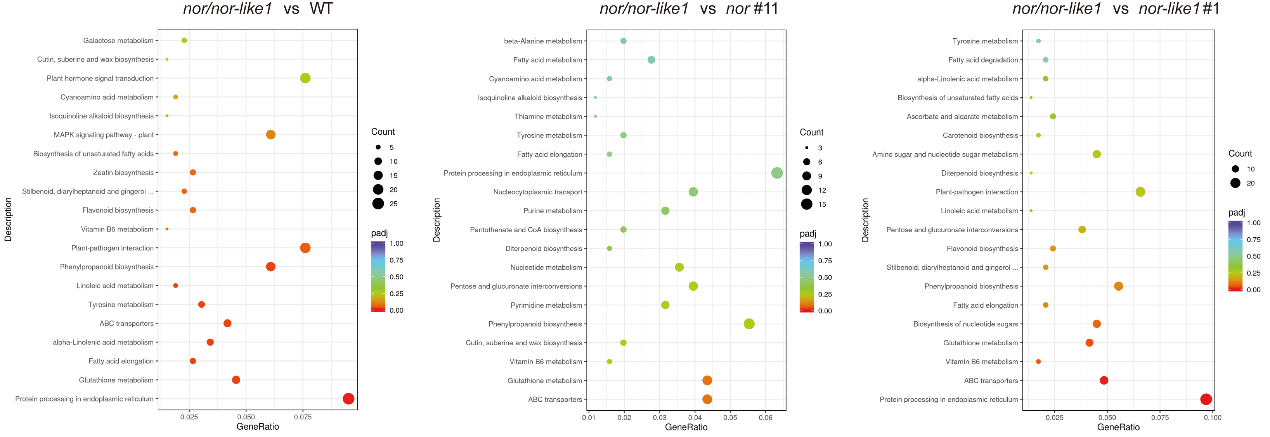


Supplementary Figure S9. Kyoto Encyclopedia of Genes and Genomes (KEGG) enrichment pathway analysis between *nor/nor-like1* stamens and WT/*nor*#11/*nor-like1*#1 stamens at MUM stage.


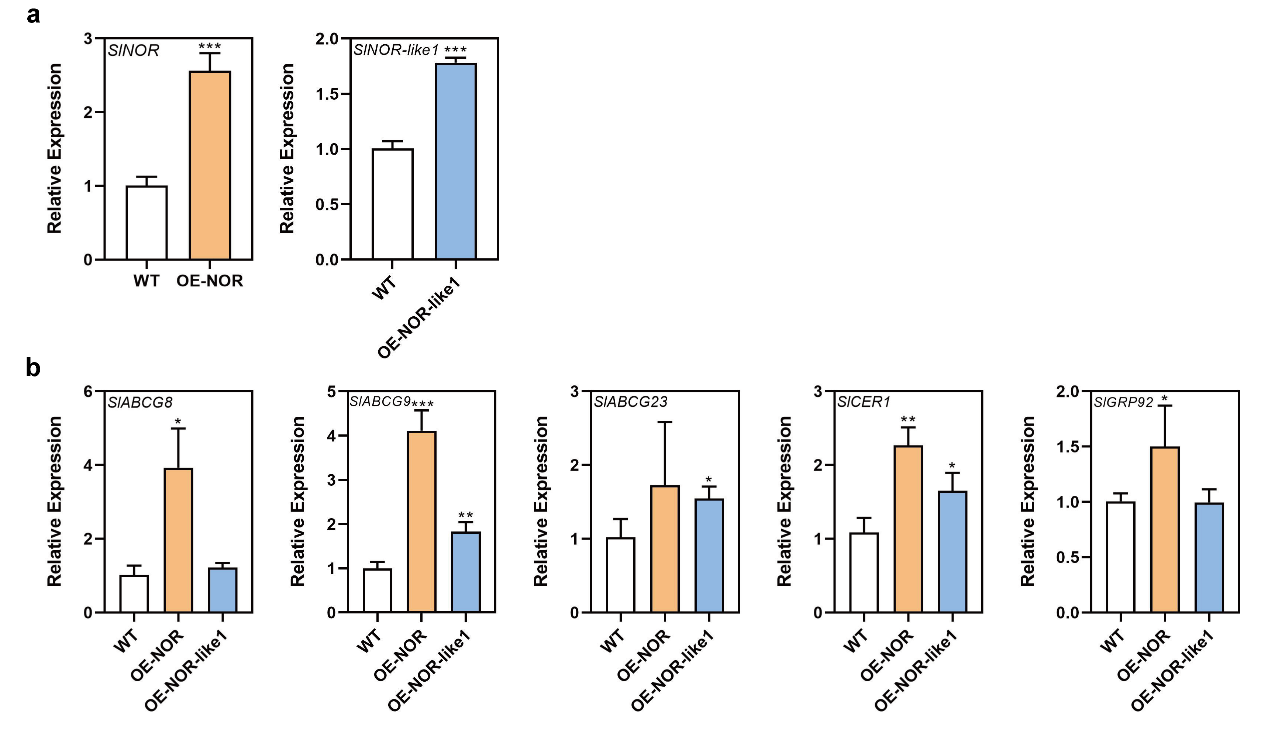


Supplementary Figure S10. The expression levels of *SlABCG8/9/23*, *SlCER1*, and *SlGRP92* in the stamens of *SlNOR* or *SlNOR-like1* over-expressing tomato plants at BM stage.

a) Over-expressing levels of *SlNOR* or *SlNOR-like1* in the (OE)-*NOR/NOR-like1* (35S promoter:: *NOR*/*NOR-like1*) stamens at BM stage. b) The expression levels of *SlABCG8/9/23*, *SlCER1*, and *SlGRP92* in the (OE)-*NOR/NOR-like1* stamens at BM stage. The error bars indicate ±SD of three biological replicates. Asterisks indicate significant differences determined by Student’s t-test (*P < 0.05, **P < 0.01, ***P < 0.001).
